# Calcitriol and TO-901317 Interact in Human Prostate Cancer LNCaP Cells

**DOI:** 10.4137/grsb.s562

**Published:** 2008-03-17

**Authors:** Jing-Huan Wang, Pentti Tuohimaa

**Affiliations:** 1 Department of Anatomy, Medical School, 33014 University of Tampere, Tampere, Finland; 2 Drug Discovery Graduate School, University of Turku, 20520 Turku, Finland; 3 Deparment of Clinical Chemistry, Tampere University Hospital, University of Tampere, 33014 Tampere, Finland; 4 Tampere Graduate School in Biomedicine and Biotechnology, University of Tampere, Tampere, Finland

**Keywords:** mRNA regulation, ABCA1, CYP24, calcitriol, LXR agonist, interaction, cell proliferation

## Abstract

Vitamin D receptor (VDR) and liver X receptor (LXR) are nuclear receptors, which regulate gene transcription upon binding of their specific ligands. VDR seems to play a role in the regulation of prostate cancer cell proliferation. ATP-binding cassette transporter A1 (ABCA1) is known to be a target gene of LXR and it has been reported to be inhibited by androgen and to be involved in the regulation of LNCaP proliferation. We find that calcitriol (1α,25(OH)_2_D_3_) inhibits both basal and a LXR agonist, TO-901317, induced ABCA1 mRNA expression but has no effect on the mRNA expression of ATP-binding cassette transporter G1 (ABCG1), LXRα nor LXRβ. TO-901317 increases both basal and calcitriol induced 25-hydroxyvitamin D_3_-24-hydroxylase (CYP24) mRNA expression and it slightly but significantly inhibits VDR mRNA expression. The inhibition of ABCA1 by calcitriol appears to be androgen-independent. Cell growth assay shows that when each of calcitriol and 5α-dihydrotestosterone (DHT) was co-treated with ABCA1 blocker, glybenclamide, cell-growth is significantly decreased compared to their own treatments respectively. Our study suggests a possible interaction between calcitriol and TO-901317 in LNCaP cells. Alike DHT, the inhibition of ABCA1 by calcitriol may be involved in its regulation of LNCaP growth.

## Introduction

Vitamin D receptor (VDR) is a ligand dependent transcription factor that belongs to a nuclear receptor family. Calcitriol (1α,25(OH)_2_D_3_) is an active form of vitamin D_3_, which mediates its biological activities through VDR. 25-hydroxyvitamin D_3_-24-hydroxylase (CYP 24) is the most sensitive vitamin D_3_ responsive gene (Wang et al. 2005; Wood et al. 2004) and thus its fluctuate expression after VDR activation has been utilized for evaluation of VDR signaling changes (Dunlop et al. 2005). It has been reported that calcitriol inhibits prostate cancer growth by androgen-dependent and androgen-independent mechanisms (Zhao et al. 2000).

Liver X receptors, LXRα and LXRβ are also ligand-dependent transcription factors and belong to the nuclear receptor family. Many of the target genes for LXRs are involved in cholesterol and fatty acid metabolism pathways. Major cholesterol-related targets of LXRs include the ATP-binding cassette transporter family members such as ABCA1, ABCG1, ABCG5 and ABCG8. ABCA1 is encoded by the gene that is mutated in Tangier disease (Brooks-Wilson et al. 1999; Rust et al. 1999), which is featured by low or absence of HDL-C and reduced total cholesterol (Serfaty-Lacrosniere et al. 1994) and is associated with increased susceptibility to atherosclerosis (Maxfield and Tabas, 2005). Interestingly, recent studies show that LXR agonist inhibits tumor growth and progression of LNCaP prostate cancer cells (Chuu et al. 2006; Fukuchi et al. 2004b) and androgenic inhibition of ABCA1 is involved in the regulation of prostate cancer growth (Fukuchi et al. 2004a).

Our previous study (Wang and Tuohimaa, 2007) suggests that LXR, VDR and androgen receptor (AR) signaling form a complex interaction in the prostate cancer LNCaP cells. In the present study, we aimed to investigate whether VDR ligand, calcitriol, had effects on the expression of LXR target gene, ABCA1, and if any, the physiological consequences concerning LNCaP cell growth; whether LXR agonist, TO-901317, had effects on the expression of VDR target gene, CYP24.

## Materials and Methods

### Reagents

1α,25(OH)_2_D_3_, was obtained from Leo Pharmaceuticals (Ballerup, Denmark). TO-901317, glybenclamide, cycloheximide (CHX) and RPMI-1640 medium were purchased from Sigma-Aldrich (Saint Louis, Missouri, U.S.A.). 5α-dihydrotestosterone (DHT) was obtained from Merck (Darmstadt, Germany). Bicalutamide was obtained from AstraZeneca (London, U.K.). FBS was purchased from Gibco-BRL (Life Technology, Paisley, Scotland). TRIzoL reagent was purchased from Invitrogen (Carlshad, U.S.A.). High Capacity DNA Archive Kit and SYBR Green PCR Master Mix Kit were purchased from Applied Biosystems (Forster City, U.S.A.).

### Cell treatment and RNA isolation

Human prostate cancer cell line LNCaP clone FGC (American Type Culture Collection) was maintained in phenol red RPMI-1640 medium, 5 ug/ml insulin and antibiotics (penicillin 100 units/ml, streptomycin 100 ug/ml) at 37 °C in a humid atmosphere with 5% CO_2_. Serum supplemented in the medium was 10% FBS otherwise 10% DCC-FBS as indicated. Cells were grown to 50% confluence and treated with vitamin D_3_ or/and other reagents. Total cellular RNA was isolated with TRIzoL reagent following the instructions from the manufacturer.

### Quantitative real-time PCR analysis and primer design

Quantitative Real-time PCR was performed in ABI PRISM 7000 Detection System (Applied Biosystems, U.S.A.). cDNA was synthesised using High Capacity Archive Kit (Applied Biosystems, U.S.A.) and was used as template for Quantitative Real-time PCR using SYBR Green PCR Master Mix kit (Applied Biosystems, U.S.A.). Primers were designed using Primer Express v2.0 software (Perkin-Elmer Applied Biosystems, Foster City, C.A.). BLASTn searches were performed to ensure that the primers were gene specific. The expression of glyceraldehyde-3-phosphate dehydrogenase (GAPDH) was used for normalization. Nucleotide sequences for primers are as following: GAPDH (**NM_002046**) Forward: 5′-CCACATCGCTCAGACACCAT-3′, Reverse: 5′-ACCAGGCGCCCAATACG-3′; ABCA1 (**NM_005502**) Forward: 5′-GAGCACCATCCGGCAGAA-3′, Reverse: 5′-CTCCGCCTTCACGTGCTT-3′; ABCG1 (**NM_207630**) Forward: 5′-GCTGCTGCCGCATCTCA-3′, Reverse: 5′-TTCCCTTCTGCCTTCATCCTT-3′; LXRα (**HSU22662**) Forward: 5′-CATGCCTACGTCTCCATCCA-3′, Reverse: 5′-CGGAGGCTCACCAGTTTCA-3′; LXRβ (**HSU07132**) Forward: 5′-GATGTCCCAGGCACTGATGA-3′, Reverse: 5′-CTGG T T C C T C T T C G G G AT C T G - 3 ′; VDR (**NM_000376**) Forward: 5′-CCTTCACCATGGACGACATG-3′, Reverse: 5′-CGGCTTTGGTCACGTCACT-3′; CYP24 (**NM_000782**) Forward: 5′-GCCCAGCCGGGAACTC-3′, Reverse: 5′-AAATACCACCATCTGAGGCGTATT-3′

### Cell growth assay

LNCaP cells were treated with hormones and/or other reagents for 0, 4 and 7 days in CellBIND 6-well plates (Sigma-Aldrich). Glybenclamide was given at a concentration which almost completely blocked the activity of ABCA1 (Becq et al. 1997). Each treatment was repeated three times. Glybenclamide was dissolved in dimethyl sulfoxide (DMSO) and all the other reagents in absolute ethanol. Control cells were treated with 0.2% ethanol plus 0.1% DMSO. Cell growth was analysed with crystal violet staining as described earlier (Kueng et al. 1989). Briefly, cells were fixed by addition of gultaraldehyde to the cell culture with a final concentration of 1% and shaking at 200 rpm for 15 min, then washed with tap water and air-dried over night. 0.1% crystal violet solution was added to stain the fixed cells for 20 min with shaking at 200 rpm. Excess dye was removed by extensive washing with tap water. The plates were air-dried over night. 10% acetic acid was used to withdraw cell-bound dye. The optical density of extracted dye was measured in 96-well plates at 590 nm by Microplate Reader (Wallac, victor 1420 multilabel counter, Turku, Finland). Final data was presented as percentage absorbance to negative control cells of corresponding treatment period.

### Statistical analysis

Data of real-time PCR are expressed as the mean values ± SD. Significance was assessed using the Student’s paired *t* test. *p < 0.05 was considered as significant, **p < 0.001 as highly significant and p > 0.05 as not significant (NS).

## Results

### Down-regulation of ABCA1 mRNA by calcitriol in LNCaP cells

**Figure 1:**
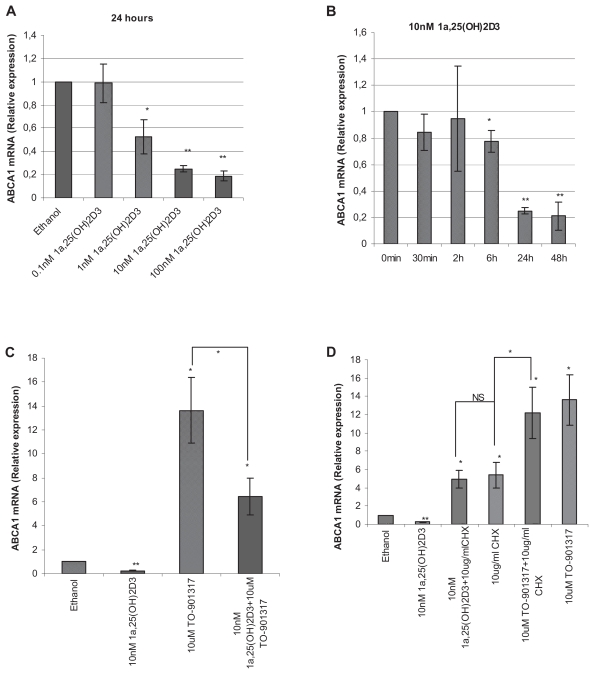
Down-regulation of ABCA1 mRNA expression by calcitriol in LNCaP cells. (**A**) LNCaP cells were treated with 0.2% ethanol and different doses of calcitriol for 24 hours or (**B**) with 0.2% ethanol and 10 nM calcitriol for different period of time. (**C**) Cells were treated with 0.2% ethanol, 10 nM 1α,25(OH)_2_D_3_, 10 uM TO-901317 and 1α,25(OH)_2_D_3_ plus TO-901317. The inhibition of ABCA1 occurred also in the presence of TO-901317. (**D**) Cells were treated with 0.2% ethanol, 10 nM 1α,25(OH)_2_D_3_, 10 ug/ml cycloheximide, 10 uM TO-901317 and 1α,25(OH)_2_D_3_/TO-901317 plus cycloheximide. Cycloheximide has no effect on TO-901317 mediated ABCA1 mRNA induction but blocked the inhibition effect of calcitriol. Relative ABCA1 mRNA expression was analysed by Quantitative Real-Time PCR (n = 3, *p < 0.05, **p < 0.001).

To test whether calcitriol has any effect on the expression of ABCA1 mRNA, prostate cancer LNCaP cells were treated with calcitriol and the mRNA expression level was analyzed by Quantitative Real-Time PCR. The results show that the expression of ABCA1 mRNA is dose dependently inhibited by 1α,25(OH)_2_D_3_ (Fig. 1A). At 10 nM, a significant inhibition of ABCA1 mRNA expression begins at 6 hours. At 24 hours, the effect of 10 nM 1α,25(OH)_2_D_3_ is at the maximum (Fig. 1B).

### Calcitriol inhibits ABCA1 mRNA induction by TO-901317

Because TO-901317 induces ABCA1 expression (Wu et al. 2004), we were interested to examine whether calcitriol had any effect on ABCA1 mRNA expression in the presence of TO-901317. Our results show that TO-901317 induces 14-fold (p = 0.0014) of ABCA1 mRNA expression and 10 nM calcitriol with 10 uM TO-901317 results in a 47% (p = 0.017) decrease of it (Fig. 1C).

### Effect of cycloheximide on calcitriol mediated inhibition of ABCA1 mRNA expression

Next we studied whether the decrease of ABCA1 mRNA effect of calcitriol was a direct effect. Our data show that in the presence of protein synthesis inhibitor, cycloheximide, calcitriol fails to decrease mRNA level of ABCA1 (Fig. 1D), suggesting an indirect regulation of ABCA1 by calcitriol. In comparison, cycloheximide has no effect on TO-901317 mediated ABCA1 mRNA induction (Fig. 1D).

### Effect of calcitriol and TO-901313 on LXRα, LXRβ and ABCG1 expression

**Figure 2:**
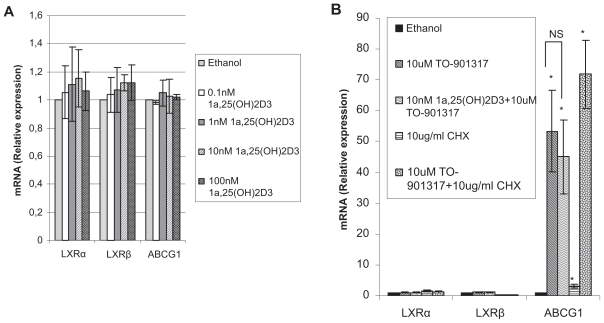
Effects on the mRNA expression of LXRα, LXRβ and ABCG1 by calcitriol, TO-901317 and cycloheximide. LNCaP cells were treated for 24 hours with 0.2% ethanol, and (**A**) different concentrations of calcitriol (**B**) 10 uM TO-901317, 10 ug/ml cycloheximide, TO-901317 plus calcitriol and/or plus cycloheximide. Calcitriol has no effect on the mRNA expression of LXRα, LXRβ and ABCG1 whether TO-901317 is present (**B**) or not (**A**). Relative mRNA expression were analysed by Quantitative Real-Time PCR (n = 3, *p < 0.05, **p < 0.001).

Alike ABCA1, ABCG1 is also a LXR target gene. Thus, we studied whether calcitriol affected the mRNA expression of ABCG1. In addition, to test whether the regulation of ABCA1 by calcitriol is via the regulation of LXRα and LXRβ, the mRNA expression of these two genes were studied as well. At 24 hours, calcitriol has no effect on any of the three genes examined at different concentrations ranging from 0.1 nM to 100 nM (Fig. 2A). In comparison, TO-901317 significantly increases 53-fold (p = 0.03) of the expression of ABCG1 mRNA (Fig. 2B). In the presence of calcitriol, TO-901317 increases ABCG1 expression 45-fold (p = 0.03) (Fig. 2B). There is no statistically significant difference between TO-901317 treatment alone and in combination with calcitriol (p = 0.58), implying calcitriol has no effect on TO-901317 induced ABCG1 expression as well. Cycloheximide can not block the induction of ABCG1 by TO-901317 (Fig. 2B).

### Effect of TO-901317 on CYP24 and VDR expression

**Figure 3:**
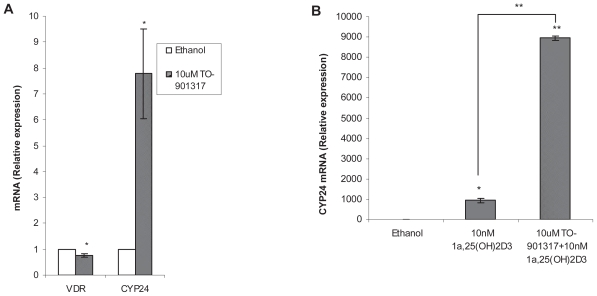
Effects on the mRNA expression of CYP24 and VDR by TO-901317. LNCaP cells were treated with 0.2% ethanol, 10 nM calcitriol and 10 uM TO-901317 alone or in combination for 24 hours. TO-901317 inhibits VDR but induces basal CYP24 expression (**A**) as well as calcitriol mediated CYP24 induction (**B**). Relative mRNA expression were analysed by Quantitative Real-Time PCR (n = 3, *p < 0.05, **p < 0.001).

Studies described above deal with the effect of VDR ligand, calcitriol, on the mRNA expression of LXRs and their target genes. Next we were interested to investigate the other way around, which was to study whether LXR agonist, TO-901317, had any effect on the mRNA expression of VDR and its target gene CYP24. Our results show TO-901317 inhibits slightly but significantly the VDR mRNA expression (Fig. 3A) and there is no statistically significant difference between the TO-901317 treatment alone or in combination with calcitriol (data not shown). TO-901317 not only increases basal CYP24 mRNA expression 8-fold (p = 0.03) (Fig. 3A), but also enhances 10-fold (p < 0.001) of calcitriol mediated induction of CYP24 expression (Fig. 3B).

### Androgen-dependency of ABCA1 expression

**Figure 4:**
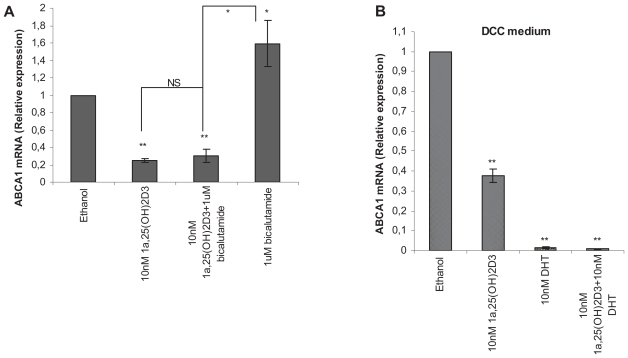
Androgen-dependency of ABCA1 expression. LNCaP cells were treated for 24 hours with 0.2% ethanol, 10 nM calcitriol, 1 uM bicalutamide and calcitriol plus bicalutamide in normal medium (**A**), or with 10 nM calcitriol, 10 nM DHT and calcitriol plus DHT in DCC medium (**B**). Relative mRNA expression was analyzed by Quantitative Real-Time PCR (n = 3, *p < 0.05, **p < 0.001).

It has been reported that androgen inhibits ABCA1 expression in LNCaP cells (Fukuchi et al. 2004a). Actions of vitamin D involve both androgen-dependent and androgen-independent mechanisms (Zhao et al. 1997; Zhao et al. 1999; Zhao et al. 2000). Thus we examined whether the inhibition of ABCA1 mRNA expression was androgen-dependent. As a positive control, androgen was included in our study. We also find that androgen inhibits the expression of ABCA1 (Fig. 4B). In the medium supplemented with the normal serum, androgen receptor antagonist, bicalutamid, causes a significant increase of ABCA1 expression (Fig. 4A). Despite the presence of bicalutamide, calcitriol decreases significantly the mRNA expression level of ABCA1 to the level with calcitriol treatment alone (Fig. 3A). Furthermore, in medium containing dextran-charcoal-stripped serum (low androgen serum), which is referred to DCC medium herein, 10 nM calcitriol still significantly decreases ABCA1 expression by 64% (p < 0.001) (Fig. 4B).

### Effect of calcitriol, TO-901317, DHT and glybenclamide on LNCaP proliferation

**Figure 5:**
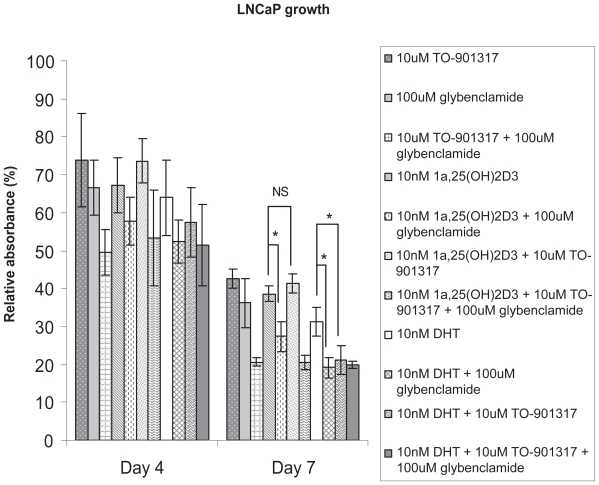
Effect of calcitriol, TO-901317, DHT and glybenclamide on LNCaP proliferation. LNCaP cells were treated for 0, 4 and 7 days (data at Day 4 and 7 are given) with 1.10 uM TO-901317; 2.100 uM glybenclamide; 3.10 uM TO-901317+100 uM glybenclamide; 4.10 nM 1α,25(OH)_2_D_3_; 5.10 nM 1α,25(OH)_2_D_3_+100 uM glybenclamide; 6.10 nM 1α,25(OH)_2_D_3_ + 10 uM TO-901317; 7.10 nM 1α,25(OH)_2_D_3_+10 uM TO-901317+100 uM glybenclamide; 8.10 nM DHT; 9.10 nM DHT+100 uM glybenclamide; 10.10 nM DHT+10 uM TO-901317; 11.10 nM DHT+10 uM TO-901317+100 uM glybenclamide. Negative control cells were treated with 0.2% ethanol + 0.1% DMSO. Crystal violet staining analysis was used to measure the absolute absorbance which reflects absolute cell number. Data shown is relative absorbance (Relative absorbance = (absolute absorbance)_compound_/(absolute absorbance)_ethanol_ * 100). Day4 and day7 treatments have the same trend for each treatment indicated. (n = 3, *p < 0.05, **p < 0.001).

Finally, we were interested to study whether the inhibition of ABCA1 by calcitriol rendered any physiological effect regarding LNCaP growth, and whether calcitriol and DHT had the same effect on LNCaP cell proliferation when they were individually co-treated with glybenclamide or TO-901317. Figure 5 shows that day4 and day7 treatments have the same trend for each treatment indicated. In comparison to the negative control, which is 100% in relative absorbance, both calcitriol and DHT decreases LNCaP cell growth. At day7, when each of calcitriol and DHT is co-treated with glybenclamide, cell growth is significantly decreased compared to their own treatment respectively. However, when co-treated with TO-901317, calcitriol shows no significant difference and DHT shows significant decrease of cell proliferation compared with each hormone treatment alone, respectively. TO-901317 and glybenclamide individually decreases LNCaP cell growth. Their co-treatment further decreases cell proliferation, which remains unchanged whether when calcitriol or DHT is added.

## Discussion

In this study, we report for the first time, that calcitriol inhibits the mRNA expression of ABCA1. Calcitriol not only decreases basal level of ABCA1 expression, but also inhibits the LXR agonist, TO-901317, mediated induction of ABCA1. On the other hand, TO-901317 alone increases CYP24 mRNA expression and, furthermore, it enhances calcitriol mediated induction of this gene. The increase of CYP24 mRNA by TO-901317 is not due to an increase of VDR. Calcitriol has no effects on LXRα and LXRβ expression, suggesting that the inhibition of ABCA1 by calcitriol is not due to the down-regulation of these two transcription factors. Previously, Takahide M et al. (Jiang et al. 2006) reported that 1α,25(OH)_2_D_3_ blunted the LXRα-mediated induction of cholesterol 7alpha-hydroxylase mRNA in H4IIE rat hepatoma cells. Cholesterol 7alpha-hydroxylase is the rate-limiting enzyme in the catabolism of cholesterol to bile acid (Hubacek and Bobkova, 2006; Shefer et al. 1970) and it is stimulated by oxysterol receptor, LXRα (Chiang et al. 2001; Peet et al. 1998). Thus, it appears that VDR negatively regulates LXR mediated induction of ABCA1 as well as cholesterol 7alpha-hydroxylase, which are all involved in cholesterol efflux and bile acid synthesis. On the other hand, we find that TO-901317 slightly but significantly inhibits VDR expression and up-regulates CYP24, which is up-regulated by VDR (Chen and DeLuca, 1995). CYP24 is an enzyme responsible for inactivating of active vitamin D metabolites such as 1,25α(OH)_2_D_3_ and 25(OH)D_3_ (Chen et al. 1993; Ohyama et al. 1993). This suggests that LXR agonist might play a role in the negative regulation of the actions of VDR ligand. Stimulated by a growing body of preclinical evidence that vitamin D inhibits the proliferation of prostate cancer, clinical trials of calcitriol in prostate cancer patients have been carried out since the 1990s (Beer, 2003). On the other hand, our present study shows that TO-901317 inhibited LNCaP cell proliferation and Liao et al. (Fukuchi et al. 2004a) find that knockdown of ABCA1 expression by RNA interference in androgen-dependent cells increased their rate of proliferation. Based on their study they proposed a potential of use of LXR signaling as a therapeutic target in prostate and other cancers (Chuu et al. 2007). Our present study suggests a mutual negative regulation of the actions of the ligands of VDR and LXR. This implies that cautions have to be taken whether which of the VDR or LXR signaling was considered for the therapeutic target in prostate cancer.

ABCA1 has been reported to be regulated by androgen in LNCaP cells (Fukuchi et al. 2004a). Our study shows in normal medium, AR antagonist, bicalutamide, increases ABCA1 level and calcitriol acts as an antagonist of bicalutamide. Furthermore, calcitriol significantly inhibits ABCA1 in low-androgen-serum containing medium. This suggests that the inhibition of ABCA1 by calcitriol is most probably androgen-independent.

ABCA1 is a cholesterol exporter (Oram and Heinecke, 2005; Venkateswaran et al. 2000). It may be involved in cell proliferation as reported earlier (Fukuchi et al. 2004a), which showed specific knock-down of ABCA1 expression by RNA interference resulted in an increase of LNCaP proliferation. The same research group (Fukuchi et al. 2004a) demonstrated that DHT inhibited ABCA1 expression and this regulation is involved in DHT mediated LNCaP proliferation. Here we find that calcitriol also inhibits the expression of ABCA1. Thus, we were interested to test whether the inhibition of ABCA1 by calcitriol had effect on cell proliferation, and we included DHT as a parallel in our cell growth assay. We did not observe an increase of cell proliferation after glybenclamide treatment as expected, given that knock-down of ABCA1 increases LNCaP proliferation (Fukuchi et al. 2004a). This might be because glybenclamide can inhibit a broad range of ABC transporters, including ABCA1 and cystic fibrosis transmembrane conductance regulator (Hasko et al. 2002). The final output of specific blocking of ABCA1 by glybenclamide thus is not seen here. This explains why co-treatment of glybenclamide and TO-901317 dose not give a cell growth level which is in between of the level from each reagent treatment alone, but it is even further decreased compared to either of the reagent treatment. Thus, here we could not draw any conclusion by comparing calcitriol treatment and calcitriol plus glybenclamide treatment. However, when each of calcitriol and DHT was co-treated with glybenclamide, cell-growth was significantly decreased compared to their own treatment respectively. This suggests that the regulation of LNCaP cell proliferation by calcitriol may involve its inhibition of ABCA1, which has some similarity to DHT, given that inhibition of ABCA1 by DHT is involved in its regulation of LNCaP proliferation (Fukuchi et al. 2004a). However, when co-treated with TO-901317, calcitriol shows no significant difference whereas DHT shows significant decrease of cell proliferation when compared with each hormone treatment alone, respectively, suggesting that DHT is more involved in LXR agonist related cell growth regulation than calcitriol does.

The inhibition of ABCA1 mRNA by calcitriol occurred not earlier than at 6 hours, indicating that the effect might be indirect. This was confirmed by using protein synthesis inhibitor, cycloheximide, which blocked calcitriol mediated decrease of ABCA1 mRNA. In comparison, it failed to block LXR agonist mediated induction of ABCA1 and ABCG1, suggesting ABCA1 and ABCG1 are directly regulated by LXR, which have been reported before (Costet et al. 2000; Karten et al. 2006). The testing of ABCA1 and ABCG1 serves as positive control in our experiments and the testing results indicate that our experimental system works normally.

In conclusion, we report for the first time that VDR ligand, calcitriol, inhibits ABCA1 mRNA expression and LXR agonist, TO-901317, induces CYP24 mRNA expression, suggesting an interaction between calcitriol and TO-901317 in prostate cancer cells, which implicates an association between VDR and LXR in prostate cancer. The inhibition of ABCA1 by calcitriol seems to be androgen-independent and might be involved in LNCaP proliferation, which has at least some similarity to DHT. Studying and understanding of the interaction between VDR and LXR/AR is critical for rational design of future clinical trials with vitamin D compounds for prevention and treatment of associated cancers such as prostate cancer.

## Abbreviations

ABCA1, G1: ATP-binding cassette transporter A1, G1
LXR: Liver X receptor
VDR: vitamin D receptor
CYP24: 25-hydroxyvitamin D_3_-24-hydroxylase
